# Meetings of Societies

**Published:** 1902-09

**Authors:** 


### MEETINGS OF SOCIETIES.

### Bristol Medico-Chirurgical Society.

*May 14th, 1902.*

Dr. Barclay J. Baron, President, in the Chair.

Mr. J. Lacy Firth showed three cases illustrating the results obtained from the **radical mastoid operation.** Case I. was that of a man, aged 27, who had had ear discharge on the right side for twenty years, and had twice had mastoid incisions made for abscess, and there had been a persistent mastoid sinus for a year. Eleven months previously the radical operation had been performed by the speaker. The skin and the meatal (conchal flap) incisions had been those advocated by Ballance, the after-treatment that by tamponading through the enlarged meatus. There had been no discharge from the ear since about six weeks after the operation, and the antro-attico-tympanal cavity was completely epidermized. The patient had had some facial paralysis after the operation, but it had rapidly and practically completely subsided. Case II. was that of a young woman of 20 who had had ear discharge for eleven years, and a persistent discharging mastoid sinus for ten years. The radical mastoid operation had been performed eleven months previously. In the operation the speaker had followed Ballance's directions in all particulars, the second or skin-grafting operation being done sixteen days after the first operation. There had been no ear discharge since about five weeks after the last operation, and the bony cavity was completely epidermized and dry and easily accessible through the enlarged meatus. The latter was not conspicuous unless closely examined. Case III. was a little girl, 8 years of age, with a history of ear discharge of five years duration. Eight months previously a mastoid abscess had been opened, and subsequently had healed. A month previously a second mastoid abscess had formed and left a sinus. The radical operation, in so far as it affected the bone, had been exactly the same as in the other two cases, and had been performed two and a half months previously. The meatus had not been incised. The after-treatment had been by tamponading, chiefly through the retro-auricular opening. It had been decided to make this opening a persistent one, and this had been accomplished. Through that opening the completely epidermized antrum was easily seen, and the tympanic chamber was now also almost completely covered with skin. Thus the drainage of the bony cavity after the operation had been a "double-barrelled" one; viz., by the retro-auricular opening and by the meatus. This method had been selected partly because the child was very nervous, and difficulty would have arisen from this in the subsequent dressing of the case if the meatus had been incised, partly because the skin over the mastoid had been extensively diseased at the time of operation. It was intended to leave the opening behind the ear unclosed for six or twelve months, until it was quite certain that suppuration in the ear had permanently ceased, and then to close it by a plastic operation. The patient could hear a whisper at 10 feet distance with the affected ear in spite of the epidermization and the absence of the membrana tympani and ossicles. Mr. Firth alluded to the improvements which had been made in recent years in the conduct of the mastoid operation. Now practically all surgeons operated on the bone itself in much the same way; that is, they aimed to throw the antro-attico-tympanal cavity into one smooth-walled cavity, which was easily drained, but in their method of incising the skin and the meatus and in the mode of treatment after the operation they differed considerably. Of the three cases he had shown each differed somewhat, in these latter respects, from the others, but the results in each had been satisfactory.—Mr. W. H. Harsant alluded to the much better results as to appearance in the two cases in which the meatus had been enlarged than in that with the retro-auricular opening. He was inclined to think that the complete mastoid operation, the conduct of which had been so much improved of late years, was being overdone. In saying this he made no reference to the cases which had been shown to this meeting. The operation seemed to be performed in cases which might be satisfactorily treated by ordinary methods. An objection to Ballance's operation was the necessity of performing a second operation under an anæsthetic.—Mr. Firth, in reply, stated that for simple chronic otorrhœa without ominous symptoms he would not perform the radical operation until he had tried all other methods of treatment worth trying and failed with them. The cases brought forward belonged to the class in which the operation was undoubtedly indicated.

Dr. Watson Williams showed a case of **acromegaly** in an adult male, which began to develop in early boyhood about the eighth year, but had apparently remained in *statu quo* for several years, and displayed none of the usual slow progressive tendency. He also demonstrated a case of **myxœdema** in a female, aged 58, which had been under treatment by thyroid gland preparations for ten years. The patient's health had been well maintained, and was better than it was when the patient had been shown to the Society nine years previously.

Mr. G. Munro Smith described a **case of loose body in the knee-joint**, and showed the patient on whom he had operated for that condition. The patient, a man aged 39, was liable to severe pain in the right knee. At the operation a portion of articular cartilage was removed which had become detached from the outer side of the internal condyle. The speaker suggested this curious lesion might be due, if it occurred in extreme flexion of the knee, to the pressure of the patella upon the outer border of the condyle; if it occurred in extension, it might be the result of extreme tension of the crucial ligament attached to the condyle. Mr. Munro Smith also showed a man upon whom he had operated for **rupture of the small intestine.** The patient, a coal miner, aged 54, had been working with a pickaxe one morning between 8 and 9 a.m., when a lump of coal fell, striking the axe and causing its handle to strike him on the abdomen near Poupart's ligament on the right side. When first seen by Mr. Smith about 11 o'clock the patient was moderately collapsed; writhing with paroxysmal pain, which radiated from the iliac region where bruising was noticed, but no wound; and had retching but no actual sickness, thoracic breathing, and an abdominal facies. At 6 p.m. a median laparotomy was performed. A rupture was discovered in the small intestine, about three-quarters of an inch long, and through this the intestinal contents, a yellowish brown fluid, were being pumped out into the peritoneal cavity. The wound was sutured with a continuous silk suture passing through all the coats, and a second row of interrupted Lembert's stitches externally. The peritoneum was mopped out and the abdomen closed. Recovery followed. Some weeks later the patient was operated upon for a hernia due to separation of the recti muscles. Forty sutures were used at the operation for the cure of this, which was successful. Rupture of the intestine, the speaker stated, was a very fatal form of accident. MacCormac had collected 13 fatal cases, and later Sieur had collected 38 cases, of which 20 recovered.—Mr. C. A. Morton inquired if the collected cases alluded to were the consecutive cases of one operator or cases collected from the journals, as in the latter case they would be too favourable and unreliable.—Dr. James Swain said that in these abdominal injuries there arose the difficulty of deciding whether there was only contusion present or whether there was a rupture of viscera. Even a slight injury might cause severe shock. If the injury was simple the shock tended to pass off, but if there was severe visceral injury it tended to increase. Another form of shock was that known as relapsing shock, in which the patient recovered for a short time and then became bad again, and this suggested hemorrhage. In the early stage, while the abdomen was still retracted, the diminution of liver dulness was a valuable sign of a ruptured hollow viscus, but later when tympanites had set in the sign was of no value.—Mr. Paul Bush thought the omission of peritoneal irrigation had been wise.—Mr. Lace related the case of a groom he had seen who had three hours previously been kicked by a horse in the right groin. There had been signs of bruising, slight rigidity below the umbilicus, and general tenderness. The man said he was feeling better than he had felt at first. It was elicited that there had been a hernia in the right groin for some years. The hernial sac was opened and found to contain a little fluid, and exploration through the neck of the sac revealed the vermiform appendix hanging by a thread, and it was removed. When the operation was nearly completed out popped a coil of intestine which had been torn completely across, but the cut edges of each segment had been for the time being closed by peritoneal adhesions, so that extravasation of bowel contents had been prevented. The edges were stuck together like those of a divided india-rubber tube. The bowel was sutured and the man recovered.—Mr. MUNRO SMITH replied that there had been moderate shock only at first, that retching had been continuous but there had been no actual vomiting, and the liver dulness had not been completely obliterated.

Mr. A. W. PRICHARD read notes on a **case of pylorectomy**, and showed the patient and the specimens obtained. The patient was a woman of 27 years of age. For nine years she had been liable to pain after eating; this had been constant for three years. Latterly vomiting had been frequent and copious, and streaks of blood had been found in the vomit. She had lost much flesh. The patient had a tubercular aspect, but no disease was found except that of the digestive organs. There was a swelling in the upper part of the abdomen extending from the ensiform cartilage to the umbilicus level. Dulness to percussion existed below the umbilicus after taking food. Laparotomy was performed, and the pylorus and neighbouring parts of the stomach found to be very much thickened, as though involved in a tumour, and these parts were therefore removed. Two hours after the operation two pints of saline fluid were transfused into a vein. The patient only vomited once after the operation. She now feels better than she has done for years. The nature of the affection was doubtful. The section surface of the indurated parts was white and looked like scirrhus, but the microscope showed that it certainly was not carcinoma. The question was whether it might not be of a tubercular and inflammatory nature, or whether it was sarcoma. The speaker had to thank Mr. Mole for valuable assistance at the operation and since.—Mr. C. A. MORTON, after examining the section, thought it might be inflammatory.—Mr. W. H. HARSANT and Mr. PAUL BUSH thought the swelling was a new growth.—Mr. MUNRO SMITH said that many of the sections showed numerous round cells grouped about the vessels. The cells might be those of a sarcoma —Dr. J. SWAIN said that at the operation the growth felt like a malignant tumour, but the history of the case negatived that view.

Mr. C. A. MORTON read a paper on a series of cases of **operation for tumours of the thyroid gland**. All the cases, ten in number, were of innocent tumours, and had produced unsymmetrical swellings in the gland. He had only operated, with perhaps one exception, because the swellings were causing distress in breathing or because the enlargement was tending to increase downwards towards the mediastinum. Nine of the patients were females. In all the cases except one the tumours had been removed. Two of the cases were of cystic adenoma. The case in which he had not removed the tumour was that of a man of 64. His tumour was cystic and of the size of an orange, and there was extensive calcification of its walls. It had been treated by incision and drainage in 1898. There had been stridor some days after the operation which must have been from spasm, not from compression of the trachea, as the insertion of an ordinary tracheotomy tube had relieved it. A sinus persisted for several years, but lately this had closed. Mr. Morton alluded to the necessity of cutting well down into the proper capsule of the tumours before attempting to enucleate them. The bleeding might be free. Examination should be made for other growths than those most obvious. The difficulty of distinguishing between a cyst and a solid adenoma was mentioned, and indicated that enucleation should be the treatment in either case.—Dr. J. Swain agreed that it was advisable to operate early on the tumours growing low down in the neck. He thought it was a good thing to allow the patient to rally from the anæsthesia before finally closing the skin wound.—Mr. Harsant asked if there had been any troublesome recurrent hemorrhage. In some recorded cases this had been very severe. Personally he felt less anxious after thyroidectomy than after the enucleation of adenomata.—Dr. Stack alluded to the plan he had seen Mr. Berry successfully adopt of very quickly enucleating the tumours and then seizing two or three points at random in the fundus of the cavity left and pulling them up and thus bringing the deep parts into view.—Mr. Paul Bush said that the cysts shelled out as easily as the solid tumours. He believed in draining the cavity left for twenty-four hours and leaving a stitch to tie later where the tube had been. There was nearly always some bleeding after enucleation.—Mr. Hancocke Wathen alluded to the apparent frequency of these thyroid swellings in the district, and asked if it was due to the water supply.—Mr. Morton replied that he had had no trouble from recurrent hemorrhage.

